# Personalized machine learning of depressed mood using wearables

**DOI:** 10.1038/s41398-021-01445-0

**Published:** 2021-06-09

**Authors:** Rutvik V. Shah, Gillian Grennan, Mariam Zafar-Khan, Fahad Alim, Sujit Dey, Dhakshin Ramanathan, Jyoti Mishra

**Affiliations:** 1 Department of Psychiatry, University of California, San Diego, CA USA; 2 Neural Engineering and Translation Labs, University of California, San Diego, CA USA; 3 Mobile Systems Design Lab, Dept. of Electrical and Computer Engineering, University of California, San Diego, CA USA; 4 Department of Mental Health, VA San Diego Medical Center, San Diego, CA USA

**Keywords:** Depression, Predictive markers

## Abstract

Depression is a multifaceted illness with large interindividual variability in clinical response to treatment. In the era of digital medicine and precision therapeutics, new personalized treatment approaches are warranted for depression. Here, we use a combination of longitudinal ecological momentary assessments of depression, neurocognitive sampling synchronized with electroencephalography, and lifestyle data from wearables to generate individualized predictions of depressed mood over a 1-month time period. This study, thus, develops a systematic pipeline for N-of-1 personalized modeling of depression using multiple modalities of data. In the models, we integrate seven types of supervised machine learning (ML) approaches for each individual, including ensemble learning and regression-based methods. All models were verified using fourfold nested cross-validation. The best-fit as benchmarked by the lowest mean absolute percentage error, was obtained by a different type of ML model for each individual, demonstrating that there is no one-size-fits-all strategy. The voting regressor, which is a composite strategy across ML models, was best performing on-average across subjects. However, the individually selected best-fit models still showed significantly less error than the voting regressor performance across subjects. For each individual’s best-fit personalized model, we further extracted top-feature predictors using Shapley statistics. Shapley values revealed distinct feature determinants of depression over time for each person ranging from co-morbid anxiety, to physical exercise, diet, momentary stress and breathing performance, sleep times, and neurocognition. In future, these personalized features can serve as targets for a personalized ML-guided, multimodal treatment strategy for depression.

## Introduction

Depression accounts for the largest national and global mental health burden and is a leading cause of disability worldwide. Overall, depression affects 16 million Americans and 322 million people worldwide^1,2^. Across the lifetime, 10% of all men and 20% of all women experience depression. For millions of sufferers who seek depression treatment, it is sadly a recurrent problem. Antidepressant medications are the first line of treatment, but they have low efficacy - only one-third of all patients show symptom remission as evidenced in large clinical trials^3,4^. As a result, over the last decade, the economic burden of depression has grown by over 20%, and is estimated at an astounding $210 billion per year^5^. Emerging evidence suggests that the COVID-19 pandemic is further exacerbating the prevalence of depression in the general population^6,7^. It is clear that more effective and scalable strategies are urgently needed for depression therapeutics.

Studies of behavioral interventions for depression in multiple lifestyle-oriented domains have shown much promise^8^. Randomized controlled studies show that better sleep hygiene^8,9^, physical activity interventions^10^, as well as mindfulness meditation^11,12^ can all benefit depressed patients. Evidence for efficacy also exists for dietary interventions that focus on reducing processed fats and sugars and moderating caffeine intake^13–17^. Unfortunately, not all interventions work for all depressed patients. Depression is a multifaceted illness with genetic, behavioral, lifestyle, and interpersonal risk factors that may express as overlapping symptoms, which in turn leads to huge interindividual variability in clinical response to the same treatments or behavioral recommendations^18–20^. For these reasons, a personalized approach for enhancing mental wellbeing in depressed patients, wherein a treatment plan is tailored to each individual patient, has been recommended for nearly a decade^21^_._ Ideally, this personalized treatment would be closed-loop and adaptive in design^22,23^, i.e., constantly providing reinforcing positive feedback and adjusting based on the individual patient’s performance and progress. Despite this clearly identified need, no research to-date has designed algorithms that would facilitate N-of-1 personalized closed-loop treatment for depression, taking into account multiple facets of individual behaviors.

Here, we leverage smartphone-based ecological momentary assessments (EMA)^24^ combined with wearable based lifestyle data on sleep, physical activity, and stress metrics, as well as neurocognitive assays on a scalable electroencephalography (EEG) platform^25^, to longitudinally ascertain the predictors of depressed mood in young adults with moderate depression symptoms. We apply machine learning models to the multidimensional data collected over a 1-month period and extract the top features that can then be used to guide personalized intervention. Notably, recent research in depression has used mobile lifestyle monitoring and/or leveraged regression/machine learning models to predict mood^26–29^. In some studies, multidimensional data have been used to choose between one of two potential treatment options for patients^20,30–33^. However, the emphasis of these past studies has been cross-sectional research. No study, to the best of our knowledge, has generated N-of-1 models that can then guide personalized multimodal treatment.

Approaches that adopt prediction models based on prior population data have some limitations. First, it is not always possible to access a sufficiently large, standardized database of already treated patients in a clinical setting. Second, these approaches are restricted to a decision between two or more fixed treatment packages, e.g., psychotherapy vs. antidepressant medications. Finally, methodological experts have argued that personalized predictions can only be made based on prior data from the individual for whom a prediction is to be made (idiographic data) and not with aggregated data from other individuals (nomothetic data)^34,35^. There is negligible research in the N-of-1 patient domain towards prediction of illness and treatment design^29,36^; any research that exists has not comprehensively taken into account multiple intervenable facets of the individual’s functioning that may determine their ill-state. Here, we hypothesized that idiographic, personalized prediction of depressed mood, leveraging ML on 1-month of continuous multidimensional lifestyle and neurocognitive data, is feasible. We aimed to not only predict depressed mood scores, but further to identify the variables (or combination of variables) that most robustly predict depression in each person, which can then be harnessed to guide person-specific depression treatment in the future.

## Materials and methods

### Participants

Overall, 14 adult human subjects (mean age 21.6 ± 2.8 years, 10 females) took part in this study. All participants were referred to the study from the University of California San Diego College Mental Health Program^37^. For study inclusion, participants must be experiencing moderate depression symptoms, which we assessed using the Patient Health Questionnaire, PHQ-9 scale (score > 9; participant score range 10–17)^38^. A structured clinical interview was not conducted for this study. Three participants on current psychotropic medications were at a stable dose 1 month prior to study initiation and agreed to maintain their stable dose throughout the course of this 1-month study. Suicidal behaviors were screened using the Columbia Suicide Severity Rating Scale^39^, and no participants demonstrated suicidal behaviors at study initiation, or as assessed every 2 weeks during the 1-month study. All participants provided written informed consent for the study protocol approved by the University of California San Diego institutional review board, UCSD IRB# 180140. All data were collected in the year prior to COVID-19 research restrictions.

### Study procedure

Participants took part in a 1-month study. On days 1, 15, and 30, participants took part in neurocognitive assessments that were synchronized with EEG^25,40^. On day 1, participants also downloaded our Unity-based *BrainE* application on their iOS/Android smartphone^40^. Within the *BrainE* app, participants accessed daily EMAs on a module called *MindLog* on which they provided mood and lifestyle ratings 4× per day for 30 days. The app sent regular notifications daily at 8 a.m., 12 p.m., 4 p.m., and 8 p.m. to all participants following the methodology of recent research on longitudinal mood monitoring^28^. In addition, on day 1, participants received a Samsung Galaxy wristwatch that they wore throughout the 30-day study, except while charging the watch for a few hours once every 2–3 days.

### Neurocognitive assessments

Participants completed six cognitive assessment games designed to assay inhibitory control, interference processing, working memory, emotion bias, internal attention, and reward processing. These assessments have been described previously and shown to have high test-retest reliability (Cronbach’s alpha ~0.8)^25,41^. Supplementary Fig. 1 shows a schematic layout of all neurocognitive assessment tasks and Supplementary Table 1 describes the variables collected from these assessments for modeling. Assessments were deployed on the Unity-based *BrainE* platform with simultaneous EEG, delivered on a Windows-10 laptop at a comfortable viewing distance. The Lab Streaming Layer (LSL) protocol was used to time-stamp all stimuli and response events in all cognitive assessments^42^. Each cognitive assessment session (on days 1, 15, and 30) lasted ~45 min. Individual assessment details are provided:

#### Assessment 1: Inhibitory control

Participants accessed a game-like task, “Go Wait”^43,44^. The basic task framework was modeled after the standard test of variables of attention^45^. In this two-block task, visual stimuli of colored rockets appeared in either the upper or lower central visual field. The task sequence consisted of a central fixation “+” cue for 500 ms, followed by a rocket stimulus of either blue target color or other iso-luminant nontarget color (brown, mauve, pink, purple, teal), presented for 100 ms. For blue rocket targets, participants were instructed to press the spacebar on the laptop keyboard as quickly as possible (“go” trials). For nontarget color rockets, participants withheld their response until the fixation “+” cue flashed briefly on the screen at 2 s post stimulus for 100 ms duration (“wait” trials). Thus, participants were required to be cognitively flexible in their responses based on the stimulus cues. Trial response feedback was provided for accuracy as a smiley or sad face emoticon presented 200 ms post response for 200 ms duration, followed by a 500 ms inter-trial interval. Both task blocks lasted 5 min and consisted of 90 trials per block with 30/60 target/nontarget ratio in block 1 and 60/30 ratio in block 2; all stimuli were presented in a shuffled order. Four practice trials preceded the first task block, and participants received a percent block accuracy score at the end of each block with a series of happy face emoticons (up to ten). All other neurocognitive assessments described below also used the same trial and block feedback specifications as in this task in order to promote task engagement. Total task time was 10 min.

#### Assessment 2: Interference processing

Participants accessed the game-like task, “Middle Fish”, which was an adaptation of the Flanker assessment^46^. Participants attended to a central fixation “+” cue for 500 ms, and then viewed an array of fish presented either in the upper or lower central visual field for 100 ms. On each trial, participants had a 1 s response window to detect the direction of the middle fish (left or right) while ignoring the flanking distractor fish that were either congruent or incongruent to the middle fish, i.e., faced the same or opposite direction to the middle fish. Overall, 50% of task trials had congruent distractors and 50% were incongruent. The task used the same trial-by-trial and end-of-block feedback procedures as described for the first inhibitory control assessment above. A brief practice of 4-trials preceded the main task of 96 trials presented over two blocks for a total task time of 8 min.

#### Assessment 3: Working memory

Participants accessed a game-like task, “Lost Star”, which was based on the visuo-spatial Sternberg task^47^. The task sequence had the participants attend to a central fixation “+” cue for 500 ms, followed by a spatially distributed test array of objects (i.e., a set of blue stars) for 1 s. Participants were required to maintain the locations of these stars for a 3 s delay period, utilizing their working memory. A probe object (a single green star of 1 s duration) was then presented in either the same spot as one of the original test stars, or in a different spot than any of the original test stars. Participants were instructed to respond whether or not the probe star had the same or different location as one of the test stars. We implemented this task at the threshold perceptual span for each individual, which was defined by the number of test star objects that the individual could correctly encode without any working memory delay. For this, a brief perceptual thresholding period preceded the main working memory task, allowing for equivalent perceptual load to be investigated across participants^48^. During thresholding, the set size of test stars increased progressively from 1 to 8 stars based on accurate performance where 100% accuracy led to an increment in set size; <100% performance led to one 4-trial repeat of the same set size and any further inaccurate performance aborted the thresholding phase. The final set size at which 100% accuracy was obtained was designated as the individual’s perceptual threshold.

Post thresholding, the working memory task consisted of 48 trials presented over 2 blocks^49^ and used the same trial-by-trial and end-of-block feedback procedures as described for the first inhibitory control assessment above. The total task duration was 6 min.

#### Assessment 4: Emotion bias

Participants accessed the game-like task, “Face Off”, adapted from studies of attentional bias in emotional contexts^50–52^. The task integrated a standardized set of culturally diverse faces from the NimStim database^53^. We used an equivalent number of male and female faces, each face with four sets of emotions, either neutral, positive (happy), negative (sad) or threatening (angry), presented on equivalent number of trials. Each task trial initiated with a central fixation “+” cue presented for 500 ms followed by an emotional face with a superimposed arrow of 300 ms duration. The arrow occurred in either the upper or lower central visual field on equal number of trials, and participants responded to the direction of the arrow (left/right) within an ensuing 1 s response window. The task used the same trial-by-trial and end-of-block feedback procedures as described for the first inhibitory control assessment above. Participants completed 144 trials presented over three equipartitioned blocks with shuffled, but equivalent number of emotion trials in each block; a practice set of 4-trials preceded the main task. The total task duration was 10 min.

#### Assessment 5: Internal attention

Participants accessed the game-like task, “Two Tap” adapted from a prior study of breath monitoring^54^. In this task, participants attended internally, specifically, they simply closed their eyes and tapped the spacebar after every two breaths. Participants were instructed to breathe naturally. The assessment duration was 5 min. There was no feedback provided on a moment-to-moment basis. At the end of the assessment, feedback was provided on consistency, i.e., percent of responses that were within one standard deviation of all responses with a series of happy face emoticons (up to 10 for 100%).

#### Assessment 6: reward processing

Participants accessed the game-like task, “Lucky Door” adapted from prior neurophysiological studies of reward processing^55–58^. Participants chose between one of two doors, either a rare gain door (RareG, probability for gains *p* = 0.3, for losses *p* = 0.7) or a rare loss door (RareL, probability for losses *p* = 0.3, for gains *p* = 0.7). Participants used the left and right arrow keys on the keyboard to make their door choice. Door choice was monitored throughout the task. The overall expected value (EV) of the choice door was varied in two separare blocks; in the “baseline” block, EVs of choice doors did not differ, while in the “experimental” block, EV was greater for the RareG door than for the RareL door. Manipulation of EV, with greater EV tied to the RareG door, allowed for investigating individual tendencies to prioritize long-term (i.e., cumulative) vs. short-term (i.e., immediate) rewards. Rewards were coin payoffs at the end of each trial (in experimental block: RareG door yielded 60 coins at *p* = 0.3 or −20 coins at *p* = 0.7 and RareL door yielded −60 coins at *p* = 0.3 and 20 coins at *p* = 0.7; in baseline block: RareG door yielded 70 coins at *p* = 0.3 or −30 coins at *p* = 0.7 and RareL door yielded −70 coins at *p* = 0.3 and 30 coins at *p* = 0.7); these specific coin payoffs ensured no EV differences between doors in the baseline block but a cumulative EV difference of 80 coins over every 10 trials in the experimental block (cumulative RareG coins: 40; RareL coins: −40). Fourty trials were presented per block and block order was randomized across participants; two practice trials preceded the main experimental/baseline blocks. Total task time was 6 min.

### Electroencephalography (EEG)

EEG data were collected in conjunction with all cognitive tasks using a 24-channel semi-dry and wireless electrode cap and SMARTING^TM^ amplifier. Signals were acquired at 500 Hz sampling frequency at 24-bit resolution. The LSL protocol was used to time-stamp EEG markers and integrate cognitive markers^42^, and files were stored in xdf format.

### Cognitive performance data

For the inhibitory control, interference processing, working memory, and emotion bias assessments, we calculated assessment consistency and efficiency metrics for each participant at each of the three time-points (days 1, 15, and 30). Consistency was calculated as 1-CV, where CV is the coefficient of variation = standard deviation of response time/mean response time. Efficiency was calculated as the signal detection sensitivity rate. Here, signal detection sensitivity, d’= z(Hits)-z(False Alarms)^59^; all d’ values were divided by max theoretical d’ of 4.65 to obtain scaled-d’ in the 0–1 range. Efficiency was then obtained as d’ x speed, where speed = log(1/response time)^60,61^.

For the working memory task, visuo-spatial working memory span (1–8) was taken as an additional performance metric. For the internal attention task, consistency was calculated similar to the other tasks; there was no efficiency metric on this task, and mean breathing time was taken as an additional performance metric. For the reward processing task, two performance metrics were computed, *gain vs. loss bias* on the baseline block; and *difference in rare gain choices* when EV differed between choices (experimental block) vs. when EV was the same between choices (baseline block).

### Neural data

A uniform processing pipeline was applied to all EEG data based on the cognitive event markers. The pipeline included data preprocessing, and cortical source localization of the EEG data filtered within relevant theta (3–7 Hz), alpha (8–12 Hz), and beta (13–30 Hz) frequency bands. EEG processing methods are detailed in our previous publication^25^.

Briefly, data preprocessing utilized the EEGLAB toolbox in MATLAB^62^. EEG data were first resampled at 250 Hz and filtered in the 1–45 Hz range to exclude ultraslow DC drifts at <1 Hz and high-frequency noise produced by muscle movements and external electrical sources at >45 Hz. EEG data were average electrode referenced and epoched to cognitive task-relevant stimuli based on the LSL time stamps, within the −1.0 to +1.0 s event time window. The data were then cleaned using the *autorej* function of EEGLAB, which automatically removes noisy trials (>5sd outliers rejected over max eight iterations). EEG data were further cleaned by excluding signals estimated to be originating from non-brain sources, such as electrooculographic, electromyographic or unknown sources, using the Sparse Bayesian learning (SBL) algorithm (https://github.com/aojeda/PEB)^63,64^. For this, cortical source localization was performed on the EEG data using the SBL algorithm. SBL is a two-step algorithm in which the first-step is equivalent to low-resolution electromagnetic tomography (LORETA)^65^. LORETA estimates sources subject to smoothness constraints, i.e., nearby sources tend to be co-activated, which may produce source estimates with a high number of false positives that are not biologically plausible. To guard against this, SBL applies sparsity constraints in the second step wherein blocks of irrelevant sources are pruned. Source space activations are then estimated and the root mean square signals are partitioned into regions of interest (ROIs) and artifact sources. ROIs are based on the standard 68 brain region Desikan-Killiany atlas^66^ using the Colin-27 head model^67^. In this process, activations from artifact sources contributing to EEG noise from non-brain sources, such as electrooculographic, electromyographic, or unknown sources, are removed to clean the EEG data. Cleaned subject-wise trial-averaged EEG data are then processed to filter signals into theta (3–7 Hz), alpha (8–12 Hz), and beta (13–30 Hz) bands, which are separately source localized in each task to estimate their underlying cortical signals. The envelope of source signals was computed in MATLAB (*envelop* function) by a spline interpolation over the local maxima separated by at least one-time sample; we used this spectral amplitude signal for all our analyses. For ease of interpretation, here, we specifically focused on cortical activity from two brain regions important for cognitive control and implicated in mood disorders—(1) the left dorsolateral prefrontal cortex (left DLPFC), and (2) the dorsal anterior cingulate cortex (dACC)^68–74^. The left DLPFC is in the left caudal middle frontal ROI in the Desikan-Killiany atlas, and dACC activity was obtained as the average of the four caudal and posterior ACC ROIs in the Desikan-Killiany atlas.

Specifically, for the inhibitory control, interference processing, working memory, and emotion bias tasks, we extracted the DLPFC and dACC peak neural signals at 100–300 ms poststimulus onset, baseline corrected for activity in the −750 to −550 ms time window prior to stimulus presentation^25^. Activity in the theta band was used in all analyses for these tasks given its relevance to cognitive control^75^.

Given that alpha band activity is most prominent for any task performed with eyes-closed, we extracted the DLPFC and dACC signal on the internal attention task in the alpha band averaged for the 2 s prior to each breath-related response.

For the reward processing task, we extracted the DLPFC and dACC signal in the theta band in the 0–500 ms post-choice period corrected for activity in the −50 to −250 ms pre-choice window. Corresponding to the *gain vs. loss bias* cognitive task metric, we used the neural signal difference for RareG vs. RareL choices on the baseline block; and corresponding to the *difference in rare gain choice* performance metric, we used the neural signal difference for RareG choices on the experimental vs. baseline block.

### MindLog EMA

Four times per day for 30 days, participants used the *MindLog* iOS/Android app, with notifications sent at 8 a.m., 12 p.m., 4 p.m., and 8 p.m. to complete the following information. At each time point, the EMA could be completed within 2 min.

#### Mood ratings

Participants rated depression and anxiety on 7-point Likert scales. For depression, participants responded to “How happy vs. sad/ depressed do you feel right now?” with the “Happy” label anchor next to score of 1 and the “Sad or Depressed” label anchor next to score of 7. For anxiety, participants responded to “How relaxed vs. anxious do you feel right now?” with the “Relaxed” label anchor next to score of 1 and the “Anxious” label anchor next to score of 7.

#### Stress assessment

Similar to the internal attention cognitive assessment, at each EMA participants completed a rapid 30-s assessment in which they were requested to tap the mobile screen after each full breath (inhale plus exhale). Recent research shows that such monitoring can serve as a basic assay of breath-focused mindfulness that is inversely related to the internally distracted/ruminative state of the individual, which is exacerbated in depression^54,76,77^. Mean breathing time and consistency data were extracted on this rapid assessment at each EMA. Across all participants’ data, we confirmed that consistency on this task was positively correlated to heart rate variability (HRV, Spearman’s *r* = 0.11, *p* = 0.002) that is a known marker for stress^78,79^; specifically, inconsistency of performance on the stress assessment was related to lower HRV, indicative of greater stress.

#### Diet reporting

At each EMA participants reported on their consumption of sugars, fats, and caffeine in the last 4 h. While diet monitoring itself can be quite sophisticated and burdensome with both subjective reports and objective tracking methodologies^80,81^, we opted for a rapid non-burdensome assessment to ensure completion over 30 days. Specifically, within the context of depression, excessive consumption of processed fats and sugars has been related to the severity of symptoms, and intervention to change such diet patterns has shown success^13–16^. Hence, based on a standard assessment of dietary fats and sugars^82^, we asked the following questions 4× per day, completed on a 0–6 item scale:

##### Fats

How many of these items have you had in the last 4 h? Red meat burger/sandwich; sausage/salami/bacon; whole egg; white bread; pizza; cheese; french fries; chips; butter popcorn; whole milk/milkshake; and fast food take-out.

##### Sugars

How many of these items have you had in the last 4 h? Cake/cookies; ice-cream; chocolate; candy; pancakes/french toast; jam/honey; soda; juice or other sweetened beverage; and cereal with added sugar.

##### Caffeine

How many servings of caffeine (coffee/tea/energy drink) have you had in the last 4 h?

### Smartwatch data

From the Samsung Galaxy wristwatch, we extracted features corresponding to (1) heart rate; (2) step count and exercise including speed, calories burned, distance, and duration; and (3) sleep duration^83^. For all features, start and end times were extracted. In addition, HRV metrics were obtained from the Tizen photoplethysmography (PPG) on the watch^84^.

### Machine learning (ML) models training and evaluation strategy

This included (1) data ingestion and feature extraction; (2) data preprocessing for ML modeling; and finally, (3) the ML model training and evaluation.

#### Data ingestion and feature extraction

The data from all the sources were carefully aggregated and stored in local storage. Raw data had different sampling frequencies—seconds to minutes for smartwatch data, hours for EMA data, and days for neurocognitive data. To reconcile these differences, all independent data variables were either aggregated or extrapolated based on their sampling frequencies to match the sampling frequency of the dependent variable, i.e., depressed mood ratings as the reference standard. The following features were, thereby, extracted:

1. Time of the day when a particular depression rating was taken: (6:00, 10:00), (10:00, 14:00), (14:00, 18:00), (18:00, 23:59);
2. Anxiety ratings, and mean breathing time and consistency of the 30-s stress assessment in each EMA were directly taken from the *MindLog* app data as these were completed at each time point when a depression rating was obtained;
3. All cognitive and neural data variables were mapped onto the nearest depression rating based on their respective time stamps.
4. Total amount of fats, sugars, and caffeine were taken in the last 24 h of each depression rating;
5. Smartwatch heart rate was taken as the mean value from a window of ±30 min around the time of each depression rating;
6. Cumulative step features were taken as the mean values from the past 12 h of each depression rating for each step feature separately;
7. Cumulative exercise features were taken as the mean values from the past 24 h of each depression rating calculated for each feature separately;
8. Number of hours slept the previous night were taken relative to each depression rating;
9. HRV from the Tizen PPG was taken as the standard deviation from a window of ±15 min around the time of each depression rating.

These features were calculated and stored separately for each subject for a total of 43 features per subject. Data were also inspected using a semi-automated method, i.e., automated and manual inspection for garbage, unusable and missing values. Manual inspection of raw data was required as data formats, variable names, and file names were different for different versions of wearables and for different mobile ecosystems used, i.e., Android and iOS.

#### Data preprocessing for ML models

This step took the data matrices from the prior step for purposes of imputation, standardization, and regularization. The preprocessing took care to not alter the data’s overall distribution at the level of each participant. For personalized models, removing missing data can create unaccountable bias and lead to low accuracy on test data. Moreover, filling missing values with fixed values, mean, mode, or median can also cause problems; when filled in place of missing data, these values can alter the original multivariate distribution, which may hinder the model from generalizing actual patterns in the training dataset. Thus, for missing data, we used a regression-based multivariate imputation scheme known as iterative imputation^85,86^. This scheme models each feature with missing values as a function of other features and uses that estimate for imputation. It does so in an iterative round-robin fashion: at each step, a feature column is designated as output y, and the other feature columns are treated as inputs X. A regressor is fit on (X, y) for known y. Then, the regressor is used to predict the missing values of y, executed for each feature in an iterative fashion.

In addition, to achieve effective preprocessing over computationally heavy ML processes, a preprocessing “pipeline object” was used. Using such an object has various advantages, including but not limited to encapsulating the preprocessing steps together, and avoiding leaking statistics from the test data into the trained model in cross-validation (CV), by ensuring that the same samples are used to train the transformers and predictors, and improving run time during parallel processing. For this study, the following preprocessing pipeline strategy was devised: (1) continuous and discrete variables were processed independently, (2) discrete variables were imputed using a “most frequent class imputer”, which is basically filling missing values with the class with highest frequency, (3) the continuous variables were further divided into two sub-parts, namely, the smartwatch plus EMA variables and neurocognition variables, (4) the smartwatch plus EMA variables were imputed using an iterative imputer (aka Multivariate Imputations via Chained Equations) discussed above, (5) the neurocognition variables were imputed using a constant imputer (imputing with a constant value) due to the coarse granularity of its data, (6) all discrete variables were regularized using an ordinal encoder which results in a single column of integers (0 to n-categories - 1) per feature, and finally (7) all continuous variables were regularized using a maximum absolute scaler, which scales and translates each feature individually with the maximum absolute value in the training set such that it does not shift or centre the data, and thereby not destroying any sparsity. The data was then ready to be deployed in the ML analysis pipeline.

#### ML pipeline

A primary step to achieving robust ML models is ensuring independence between training and test and providing transparency on the models that are evaluated. The personalized ML pipeline included hyperparameter tuning, model training, evaluation, and model selection. On the one hand, ensuring independence between data, which is used for hyperparameter tuning, training and testing makes the model less prone to overfitting, and prevents the introduction of bias into the model. However, ensuring independence between training and test datasets is a particular challenge for this N-of-1 modeling project. On average, 93 ± 30 of 120 total *MindLog* EMAs were completed per participant, thus only this many data points were available for ML training and testing. A traditional k-fold CV scheme cannot be used in this case as the model performance will then be highly dependent on the small number of examples set aside for testing. Thus, to tackle this technical challenge of dealing with a small dataset and achieving a model practically free from bias and immune to overfitting, a nested CV scheme was used, with the only downside being increased computation cost and time^87,88^. Here, we specifically used a repeated fourfold CV scheme with ten repeats as the inner CV strategy and a simple fourfold CV scheme as the outer CV strategy for the overall nested CV scheme. More details on the nested CV algorithm are provided in Supplementary Methods.

**Fig. 1:**
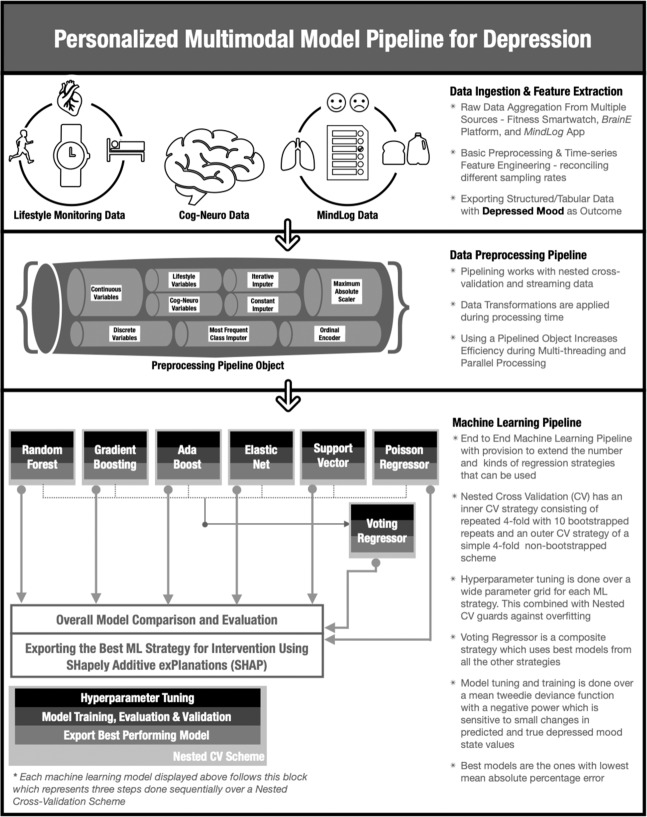
Summary of the three main steps involved in the personalized depression modeling pipeline, namely, data ingestion and feature extraction, data preprocessing, and machine learning-based analysis. These steps were carried out separately for each subject and personalized performance reports, prediction reports, and feature importance reports were obtained.

We modeled individual depressed mood ratings using the various modalities of data i.e., neurocognitive data, *MindLog* EMA data and smartwatch lifestyle data employing supervised ML regression models hyperparameter tuned and trained over the nested CV scheme. Figure 1 shows the main steps of the pipeline; the pipeline compared multiple ML strategies for each subject including random forest, gradient boost, adaptive (Ada) boost, elastic net, support vector, and poisson regressor. The voting regressor was also used that employs the best model from all the other strategies. Details on each ML strategy are provided in Supplementary Methods. After hyperparameter tuning and training over all these ML models, results were evaluated for each model, and each subject over the regression metrics of mean absolute percentage error (MAPE) and mean absolute error (MAE). We used MAPE as the performance metric to choose the best model (with lowest error) for each ML strategy^89^. MAPE is calculated using the formula:

$${\mathrm{MAPE}} = \frac{1}{{\mathrm{n}}}\mathop {\sum}\limits_{{\mathrm{k}} = 1}^{\mathrm{n}} {\left|\frac{{{\mathrm{P}}_{\mathrm{k}} - {\mathrm{A}}_{\mathrm{k}}}}{{{\mathrm{A}}_{\mathrm{k}}}}\right|} \times 100$$

where P_k_ is the predicted value of kth data point, A_k_ is the actual value of kth data point and n is the total number of data points.

The best model for each strategy was then fed in the voting regressor and the best model from this strategy was also calculated in the same manner as the other strategies. At this point, we obtained the best models for all the seven ML strategies, namely, elastic net, random forest, gradient boosted trees, Ada boosted trees, poisson regressor, support vector regressor, and voting regressor for each person. We then compared the outcome of the best performing models from each strategy and calculated the overall best model with the least overall MAPE; we chose this particular model to represent each participant (Table 1). Thus, each study participant had their own personalized model predicting their depressed mood.

**Table 1:** Summary of the performance of each personalized ML strategy conducted individually in subjects.

Subject ID	Model	Mean absolute % error		Mean absolute error		Subject ID	Model	Mean absolute % error		Mean absolute error	
		Mean	Std	Mean	Std			Mean	Std	Mean	Std
**P-1**	ab	10.07%	4.40%	0.449	0.258	**P-20**	ab	37.10%	12.80%	1.142	0.527
	en	11.89%	5.47%	0.528	0.243		en	31.67%	8.31%	1.086	0.494
	gb	10.35%	4.87%	0.477	0.264		gb	35.57%	11.42%	1.123	0.415
	pr	11.90%	4.80%	0.529	0.234		**pr**	**31.55%**	**6.22%**	**1.055**	**0.485**
	rf	9.61%	5.24%	0.440	0.276		rf	39.88%	10.57%	1.263	0.416
	**sv**	**7.55%**	**5.55%**	**0.358**	**0.291**		sv	31.86%	5.14%	1.099	0.481
	vr	9.86%	4.66%	0.447	0.239		vr	31.76%	8.25%	1.036	0.483
**P-10**	**ab**	**25.45%**	**10.13%**	**0.900**	**0.248**	**P-21**	ab	33.70%	12.98%	0.689	0.211
	en	30.70%	12.36%	1.184	0.345		en	35.45%	4.52%	0.740	0.110
	gb	32.93%	10.09%	1.235	0.285		**gb**	**33.28%**	**11.59%**	**0.824**	**0.372**
	pr	30.89%	11.65%	1.192	0.356		pr	43.88%	6.55%	0.841	0.164
	rf	26.37%	10.90%	0.973	0.390		rf	33.36%	11.39%	0.681	0.191
	sv	32.45%	14.07%	1.226	0.243		sv	39.32%	6.29%	0.815	0.089
	vr	28.16%	11.25%	1.022	0.363		vr	33.91%	7.12%	0.714	0.170
**P-12**	ab	35.13%	18.09%	0.870	0.314	**P-23**	ab	36.31%	11.84%	0.890	0.140
	en	28.05%	15.08%	0.720	0.362		**en**	**35.12%**	**15.30%**	**0.812**	**0.167**
	gb	33.80%	15.22%	0.810	0.183		gb	36.72%	13.33%	0.890	0.132
	**pr**	**26.27%**	**14.44%**	**0.650**	**0.330**		pr	37.07%	13.44%	0.812	0.136
	rf	30.77%	18.73%	0.720	0.381		rf	39.51%	12.21%	0.910	0.094
	sv	27.07%	14.39%	0.670	0.274		sv	39.26%	15.27%	0.851	0.166
	vr	26.40%	14.45%	0.650	0.302		vr	35.04%	13.93%	0.793	0.137
**P-14**	ab	46.75%	15.46%	1.063	0.235	**P-24**	ab	16.40%	12.34%	0.308	0.239
	en	55.68%	26.28%	1.264	0.411		en	13.35%	7.08%	0.258	0.235
	gb	53.03%	25.49%	1.122	0.485		gb	33.07%	14.74%	0.475	0.134
	pr	71.25%	42.99%	1.458	0.546		pr	12.10%	6.45%	0.250	0.238
	**rf**	**40.88%**	**11.87%**	**1.007**	**0.335**		rf	20.57%	20.28%	0.350	0.238
	sv	62.51%	18.54%	1.326	0.315		**sv**	**6.40%**	**6.91%**	**0.208**	**0.267**
	vr	42.73%	11.13%	0.979	0.352		vr	12.24%	2.79%	0.267	0.226
**P-15**	ab	11.42%	5.94%	0.413	0.160	**P-26**	ab	41.42%	12.73%	1.188	0.250
	en	12.73%	2.67%	0.456	0.077		en	38.21%	9.77%	1.134	0.168
	gb	12.33%	1.99%	0.445	0.011		gb	40.33%	12.33%	1.214	0.220
	pr	12.35%	2.91%	0.435	0.099		pr	39.36%	10.18%	1.161	0.161
	rf	12.04%	4.59%	0.434	0.118		rf	38.91%	6.60%	1.152	0.122
	**sv**	**10.24%**	**2.53%**	**0.378**	**0.088**		**sv**	**36.41%**	**9.63%**	**1.152**	**0.217**
	vr	11.71%	3.04%	0.422	0.105		vr	36.52%	9.75%	1.080	0.201
**P-18**	ab	30.50%	4.52%	1.153	0.158	**P-28**	**ab**	**21.23%**	**7.56%**	**0.657**	**0.131**
	**en**	**24.05%**	**11.80%**	**0.882**	**0.356**		en	28.80%	12.35%	0.906	0.426
	gb	25.80%	6.21%	0.948	0.197		gb	22.76%	11.01%	0.715	0.297
	pr	24.75%	11.62%	0.910	0.337		pr	28.23%	9.39%	0.886	0.326
	rf	26.60%	11.71%	1.000	0.340		rf	21.60%	6.84%	0.666	0.168
	sv	28.53%	6.46%	1.069	0.276		sv	29.04%	14.43%	0.896	0.511
	vr	24.05%	11.80%	0.882	0.356		vr	22.36%	4.72%	0.666	0.087
**P-19**	ab	32.60%	4.62%	0.728	0.234	**P-29**	ab	77.64%	38.75%	1.319	0.282
	en	30.48%	9.46%	0.711	0.217		en	75.14%	29.31%	1.392	0.095
	gb	47.38%	5.89%	1.002	0.325		gb	64.27%	20.17%	1.245	0.182
	pr	34.35%	9.95%	0.754	0.143		pr	71.10%	27.32%	1.410	0.040
	rf	30.47%	3.24%	0.745	0.294		**rf**	**63.14%**	**26.13%**	**1.274**	**0.322**
	**sv**	**29.11%**	**6.24%**	**0.651**	**0.202**		sv	79.64%	39.65%	1.375	0.244
	vr	29.26%	5.04%	0.686	0.270		vr	71.83%	30.21%	1.289	0.207

The best performing models for each subject are highlighted. Performance metrics of mean absolute percentage error and mean absolute error are shown. Seven different ML models were used in each subject: Adaboost regressor (ab), elastic net (en), gradient boosting tree regressor (gb), poisson regressor (pr), random forest regressor (rf), support vector machine regressor (sv), and voting regressor (vr).

#### Personalized ML feature importance

We used the SHapley Additive exPlanations (SHAP), which is a game theory-based algorithm that can be used to explain feature importance for any fitted ML model^90^. SHAP is based on the principle that a prediction can be explained by assuming that each feature value of the instance is a “player” in a game where the prediction is the “payout”. It uses coalitional game theory principles to calculate how to distribute the payout among the features equitably. The Shapley value assigns payouts to players depending on their contribution to the total payout. Players cooperate in a coalition and receive a certain profit from this cooperation. The “game” is the prediction task for a single instance of the dataset. The “gain” is the actual prediction for this instance, minus the average prediction for all instances. The “players” are the feature values of the instance that collaborate to receive the gain (=predict a certain value, in this case, for each instance of depressed mood).

We calculated the Shapley value for each feature in the best-fit personalized ML model for each participant; this value is the (weighted) average marginal contribution of a feature across all possible coalitions. We replaced the feature values of features that are not in a coalition with random feature values from the dataset to get a prediction from the ML model. The computation time increases exponentially with the number of features; hence to keep the computation time manageable we used a method known as permutation Shapley explainer which approximates the Shapley values by iterating through permutations of the inputs. This is a model agnostic explainer that guarantees local accuracy (additivity) by iterating completely through an entire permutation of the features in both forward and reverse directions. One such iteration calculates exact SHAP values for the model with up to second-order interaction effects. Now, multiple iterations over many random permutations gives better SHAP value estimates for the model with higher-order interactions. We, thereby, estimated the Shapley values for all features to obtain a complete distribution of the prediction (minus the average) among the feature values. Features with large absolute Shapley values are essential, hence, we averaged the absolute Shapley values per feature across the data, rank-sorted these and then plotted the top-five rank Shapley values for each participant (Fig. 4); the goal of future studies would be to intervene on these top ML-based features individualized to each depressed patient.

## Results

The ML pipeline was executed separately in each of the 14 subjects to predict individual depression as per Fig. 1. There were up to 43 features for each subject (Supplementary Table 1) modeled across the domains of neuro-cogition, anxiety ratings concomitant with the depression ratings, instantaneous stress and breathing assessments, as well as lifestyle data including diet, sleep, and physical activity collated for the 24-h prior to each depression rating, acquired from EMAs and smartwatches.

Table 1 shows the MAPE and MAE of the best models from each ML strategy and the overall best-fit model chosen for each subject based on the lowest absolute MAPE amongst models. The predicted data were generated over a fourfold nested CV scheme wherein threefolds were used to fit the chosen hyperparameter tuned model, and onefold was used for predictions as a test set; this was repeated for all the different combinations of 3:1 train to test splits, and the results were then collated. We observed that the overall best-fit ML model varied across subjects. Ensemble learning models had best outcomes for five subjects (i.e., including Adaboost, Random Forest Or Gradient Boost), while linear models outperformed ensemble ML algorithms for the nine other subjects (i.e., including elastic net, poisson regressor, and support vector machine). We did not observe there to be any one-size-fits-all ML strategy. On average across all subjects and all models, we observed a MAPE of 27.9 ± 10.3% that corresponded to a MAE of 0.77 ± 0.27 points on the 7-point Likert scale. Of note, MAPE values appear high while MAE values are low because depressed mood was discretely modeled on a 1–7 scale, so a 1-point difference between actual and predicted outcomes would correspond to a 100% difference in MAPE.

If one were to compare by type of model, then the average MAPE across subjects was lowest for the voting regressor, 29.7 ± 9.9% with a MAE of 0.78 ± 0.25. The voting regressor is a composite strategy that chooses the best model from all other strategies. Hence, it is logical that on-average the voting regressor produced the best results, though not necessarily at the individual level, which we confirmed by a significant difference between outcomes for the individual best-fit model with lowest MAPE vs. voting regressor (MAPE difference: −1.80 ± 0.68%, *t*(13) = −2.64, *p* = 0.02). Also, given that the voting regressor chooses the best strategy amongst all other strategies, its run-time complexity assumes that other models are already computed, and is not a time-saver over executing the full ML pipeline.

**Fig. 2:**
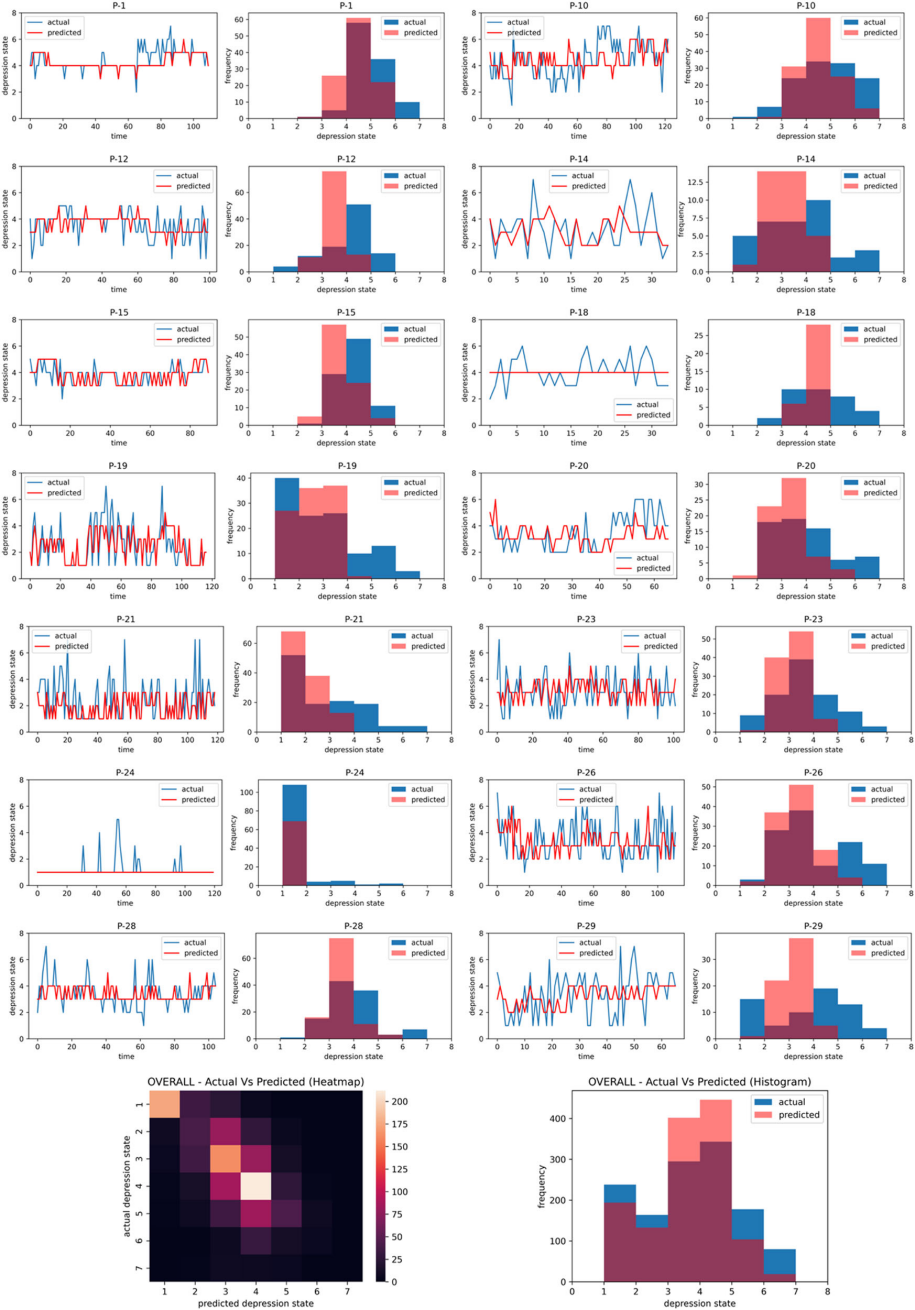
Comparisons of actual depression states as self-reported by participants vs. their predicted depression states obtained from the personalized ML pipeline with fourfold CV. Actual and predicted value comparisons are shown over time with each EMA serving as one time-step, and also compared as per their histogram distributions. The bottom row plots show the heatmap and histogram comparisons for actual vs. predicted values across all subjects.

**Fig. 3:**
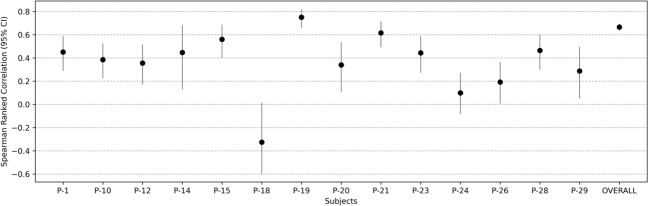
Spearman rank correlation coefficients with 95% confidence interval bounds are plotted for the relationship between the predicted and observed depressed state values over time in each individual. The overall correlation obtained by concatenating the actual vs. predicted values across all subjects, is also shown as the last data point. All correlations were significant except in P-18 and P-24. Actual correlation values are shown in Supplementary Table 2.

Figure 2 augments the performance results summarized in Table 1 in that it compares the actual values of the depression ratings with the predicted values from the best-fit ML model for each subject. Figure 2 shows two kinds of comparisons; actual vs. predicted depressed state comparisons with time where each depression rating (at each *MindLog* EMA occuring 4× daily) was one time-step, as well as the comparison between the actual and predicted value distributions in each subject. These plots show high similarity between the actual and predicted value time series and distributions. Indeed significant correlations were obtained between actual and predicted depressed ratings in most subjects, as seen in Fig. 3 (exact correlation values and associated confidence intervals and p-values are provided in Supplementary Table 2). The overall actual vs. predicted correlation across all subjects, obtained by concatenating these data values across participants, is shown as the last data point in Fig. 3 (Spearman’s rho (df, 1297) = 0.67, 95% CI [0.63 0.69], *p* < 0.0001).

From Fig. 3, it can be observed that two subjects did not show significant actual vs. predicted correlation, specifically P-18 and P-24. The inadequacy of the personalized model in these two cases was because of insufficient data for P-18 (only ~30 EMA points at which depressed state was captured as seen in Fig. 2), and insufficient variability in the data in P-24 (this participant chose scale option 1 in the large majority of cases as seen in Fig. 2). Overall, we did not find that the models significantly under or overestimated the predictions (% under-estimation = 28.38 ± 2.42%; % over-estimation = 25.05 ± 2.83%; signed-rank test, *p* = 0.17).

**Fig. 4:**
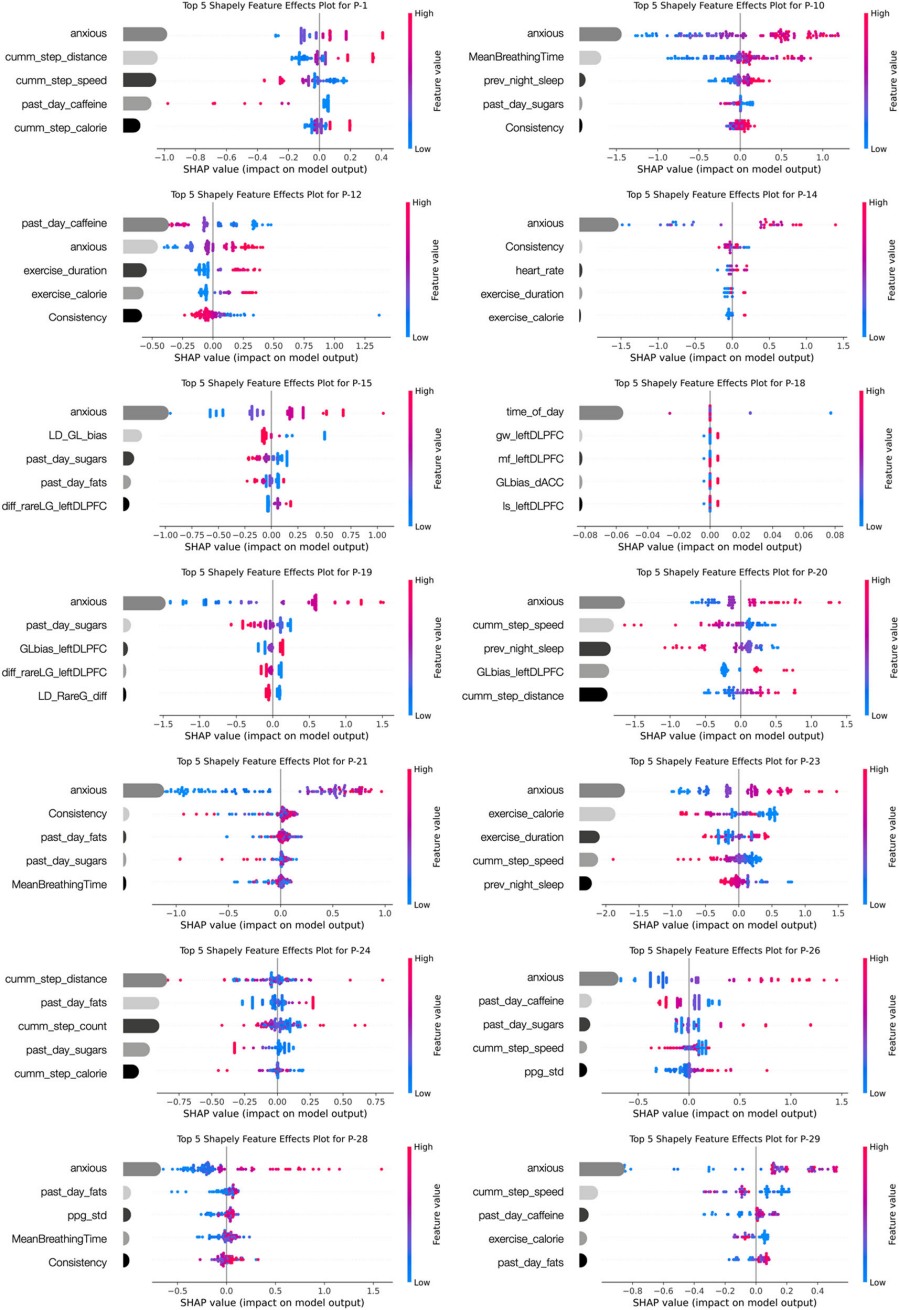
SHapley additive exPlanations (SHAP) summary plots for each subject showing rank feature importance and the feature effects. The feature importance is depicted by the size of the gray bars that represent mean absolute Shapley values for the top-five features; bar colors simply represent different feature identity. The feature effects are depicted by each colored point on the summary plot which is a Shapley value for a feature and an instance. The position on the *y*-axis is determined by the feature and on the *x*-axis by the Shapley value. The color represents the value of the feature from low (blue) to high (pink). Overlapping points are jittered on the *y*-axis direction, so we get a sense of the distribution of the Shapley values per feature. The features are ordered according to their importance. In most cases, EMA ratings of co-morbid anxiety (“anxious”) best predicted the depressed state. These plots reveal how each individual had different modalities of data as their top-rank predictors, which can then be leveraged for personalized intervention in future studies. Top variables observed were *cumm_step_distance/speed/calories/count* that depicted the cumulative step features in the past 12 h; *Mean Breathing Time* and *Consistency* that were obtained from the 30-s active stress assessment at each EMA, *prev_night_sleep* or hours of previous night’s sleep; *past day sugars/fats/caffeine*; *exercise_duration/calories* over the past 24 h; *heart rate* within the 30 min window of the EMA; *ppg_std* that depicted the HRV in the 15 min window of the EMA and *time of day*. In some cases, neurocognitive metrics also emerged as top-ranking features, including *LD_GL_bias* and *LD_RareG_diff* that respectively represented the bias towards frequent gain vs. loss in the reward task and the preference for rare gain choices when they have greater vs. equal expected value in the reward task; *GLbias_dACC/left DLPFC* that was the neural activity in the dACC/left DLPFC brain region corresponding to bias for frequent gains vs. losses on the reward task; *diff_rareLG_leftDLPFC* that was the neural activity in the left DLPFC brain region evoked to choices made on the reward task with a contrast of expected values; *gw_leftDLPFC* that was the neural activity in the left DLPFC brain region evoked to the *Go Wait* inhibitory control task; *mf_leftDLPFC* that was the neural activity in the left DLPFC brain region evoked to the *Middle fish* interference processing task; and *ls_leftDLPFC* that was the neural activity in the left DLPFC brain region evoked to the *Lost Star* working memory task.

We then computed Shapley statistics for each feature in the best-fit personalized ML model for each participant to better interpret the ML model results; Shapley values are a benchmark method for model interpretability^91^. Figure 4 shows the SHAP summary plot for each subject for the top-five ranking most important features. Both feature rank importance and feature effects are shown; each colored point on the feature effect plot is a Shapley value for the corresponding feature and an instance of the depressed state rating. These plots show how the feature predictors are personalized to each subject with unique modalities for future intervention. For instance, let us consider the predictions for P-12; caffeine intake in the last 24 h is the most prominent indicator of depression according to the summary plot. We can also see the sign of prediction, that is, the higher the feature value, the lower is the SHAP value, and hence higher overall caffeine intake is associated with better mood for this particular subject. A caution to note is that these plots show association, but not causation, between features and depressed mood. Notably, for lifestyle features of diet, exercise and sleep, we took temporality into account in the models for better interpretability i.e., these features were calculated for the 24 h prior to each depression ratings so that directionality could be understood as lifestyle prior to current mood but not vice versa.

**Fig. 5:**
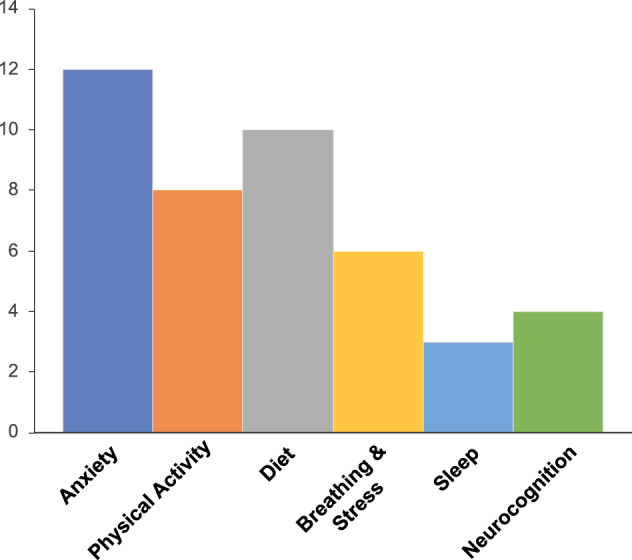
Personalized ML informed top-five ranking features across individuals. Frequency of top-five ranking Shapley feature domains across participants cumulated based on the personalized ML models in individual subjects are shown.

Overall, as expected, we found co-morbid anxiety to be highly predictive of depressed mood. Beyond this, depressed states in different individuals indeed had different predictors, making a case for personalized intervention combining multiple modalities of treatment. Figure 5 plots the frequency of different feature predictor domains for depression across participants: anxiety ratings were the top predictor in 86% of cases; physical activity over the past day including both steps and exercise based smartwatch features were top predictors in 57% cases; depression ratings were sensitive to diet including sugars, fats, and caffeine in 71% cases; the breathing and stress assessment revealed depression sensitivity in 43% participants; sleep duration was a top predictor of depression in 21% cases, and neurocognitive features particularly related to rewards processing were significant in 29% participants.

## Discussion

Depression has an incredibly large global healthcare burden^1,2^. Yet, current first-line treatments, such as antidepressants and even neuromodulation i.e., FDA-approved transcranial magnetic stimulation show low to moderate efficacy in large clinical trials^3,4,92^. In the 21st century, personalized medicine has been recommended for depression treatment^8,30,93^, but the challenge remains how to design such a strategy. Here, we present a machine learning-based personalized approach that comprehensively takes into account several factors related to the individual’s subjective symptoms, lifestyle factors, such as exercise and sleep, dietary factors, stress, and breathing based assessments, as well as cognitive function data with associated neural activations, to generate N-of-1 personalized models for individuals with depression. We further parse the personalized ML pipeline for its top-feature predictors in each individual, revealing distinct feature determinants of depression over time. Notably all features incorporated in these N-of-1 models can serve as targets for intervention. Hence, the outcomes of the personalized models can be used to design individualized interventions with a uni- or multi-feature based, i.e., personalized multimodal treatment strategy.

Here, we collected EMA app and smartwatch-based data from all participants over a 1-month time period. Further, individuals participated in EEG synchronized neurocognitive assessments at beginning, mid, and end of the study. All of these data were preprocessed and collated for the ML models within a robust pipeline. Time series feature engineering was applied to reconcile different sampling rates. Each individual’s pipeline used multiple ML strategies including ensemble learning methods of random forest, gradient boost, Adaboost as well as linear methods of elastic net regression, support vector machine, and poisson regression. A voting regressor was also employed, which is a composite strategy that selects the best model from the other strategies. To prevent overfitting, all models underwent hyperparameter tuning and nested CV. The best of seven models was selected for each individual using the MAPE criterion. Shapley feature values were then extracted for the top-five ranking features. We hereby abbreviate this approach as the personalized mental health modeling (PMHM) method, which can then be used to inform specific interventions for each individual patient. Hence, our future research will focus on applying individual interventions as directed by the PMHM features.

Notably, in previous personalized ML research from our team, blood pressure measurements were modeled using smartwatch data over 1–3 months in pre-hypertensive patients, and specific health recommendations were provided for intervention to the patients based on top-ranking model features^89^. The researchers showed significant change in blood pressure as a result of the top-feature recommendations. Thus, in future, such personalized treatment guidance can be extended for depressed individuals. PMHM can direct multimodal intervention, which encompasses evidence-based lifestyle-oriented approaches including modification of physical activity^10^, diet^13–17^, sleep hygiene^8,9^ and mindfulness meditation^11,12^. Notably, the mindful meditation intervention may also target the highly frequent anxiety feature in our models^94^. Finally, neurocognitive features can also be targeted using neuromodulation and cognitive training for depression^95–99^. Ultimately, the PMHM approach has the potential to guide N-of-1 intervention in depression, integrating aspects of lifestyle with neurocognitive stimulation. Such an integrated personalized strategy that moves away from the standard one-size-fits-all approach, has been recommended by clinicians for more than a decade, but never designed^21^. Digital medicine and the closed-loop adaptive design framework^22,23^ has an important role to play in this personalized intervention implementation, given that adherence to multiple features may need to be monitored through the course of treatment. Delivery of such a personalized intervention will form the focus of future work.

Our research differs from prior approaches in that we follow a purely idiographic approach, based on the individual subject’s data alone. All prior approaches have made use of nomothetic models that are based on aggregate data from several participants^26–29^. Modeling on multimodal cross-sectional data has previously been used to choose one of two potential treatment options for patients^20,30–33^ or to design a behavioral therapy task sequence^36^. Yet, methodological experts recommend that personalized predictions can only be made based on prior data from that individual, i.e., idiographic data^34,35^. To the best of our knowledge, this is the first study to implement such an N-of-1 model for depression, which further informs treatment. In future, as the sample data size expands across all modalities acquired in this study, it would be useful to test combinations of nomothetic and idiographic approaches.

Our study is limited in that we do not yet know the interventional utility of our N-of-1 modeling results, i.e., whether the top-feature predictors of individual depression will also serve as the best markers to engage in treatment. The models are also limited by the quality and quantity of data. We observed poor model fits for two participants, one that had minimal data and the other that had low variability in the data. Continual motivation and engagement is a core component of digital studies that we aim to iteratively improve upon. The type of sensors used also limit the results, in this case smartwatch and wireless EEG were used, and other studies may use different sensor combinations with different data variables and sampling granularity. The sampling resolution of the response variable, in this case, depression ratings collected 4× daily, is also important; while greater sampling granularity may generate different results, we did not opt for >4× per day sampling because of the longitudinal burden of the protocol. Studies designed for depressed individuals need to be cognizant of potential behavioral activation problems, and high-burden studies over long time periods may result in drop-out^100,101^; in our case no drop-out was observed. Finally, the goal of this study was to generate a personalized ML pipeline to predict depressed mood and show its feasibility; as such the study is limited by small participant sample size; restricted age range of study participants; depression assessed on self-report symptom scales but not using structure clinical diagnostic interviews; and non-exclusion of participants on stable psychotropic medications—all of these characteristics currently limit the generalizability of the results.

Depression is a multifaceted illness with several risk factors ranging from genetics, behavioral, and lifestyle factors; these risk factors may express as overlapping symptoms that ultimately result in significant interindividual variability in clinical remission and response to the same treatments^18–20^. While this individual variability is not beneficial to standard treatment studies, it can be tapped by personalized treatment protocols. Here we present a digital data-driven approach to sample several modalities of individual function that can be used to develop idiographic personalized models of depression. This PMHM approach can be leveraged in future for the implementation of novel personalized treatment, and in principle, can also be extended to enhance the prediction of other mental/physical health variables.

## Supplementary information

Supplementary Material

## Data Availability

Analytics code is available upon request from the corresponding author.
