# Supplementary material for: Personalized machine learning of depressed mood using wearables
Source: Transl Psychiatry. 2021 Jun 9;11:338. doi: 10.1038/s41398-021-01445-0 (PMC8187630; doi:10.1038/s41398-021-01445-0)
Supplement: Supplementary file 1 — Supplementary Material [file 41398_2021_1445_MOESM1_ESM.pdf]

Supplementary Materials

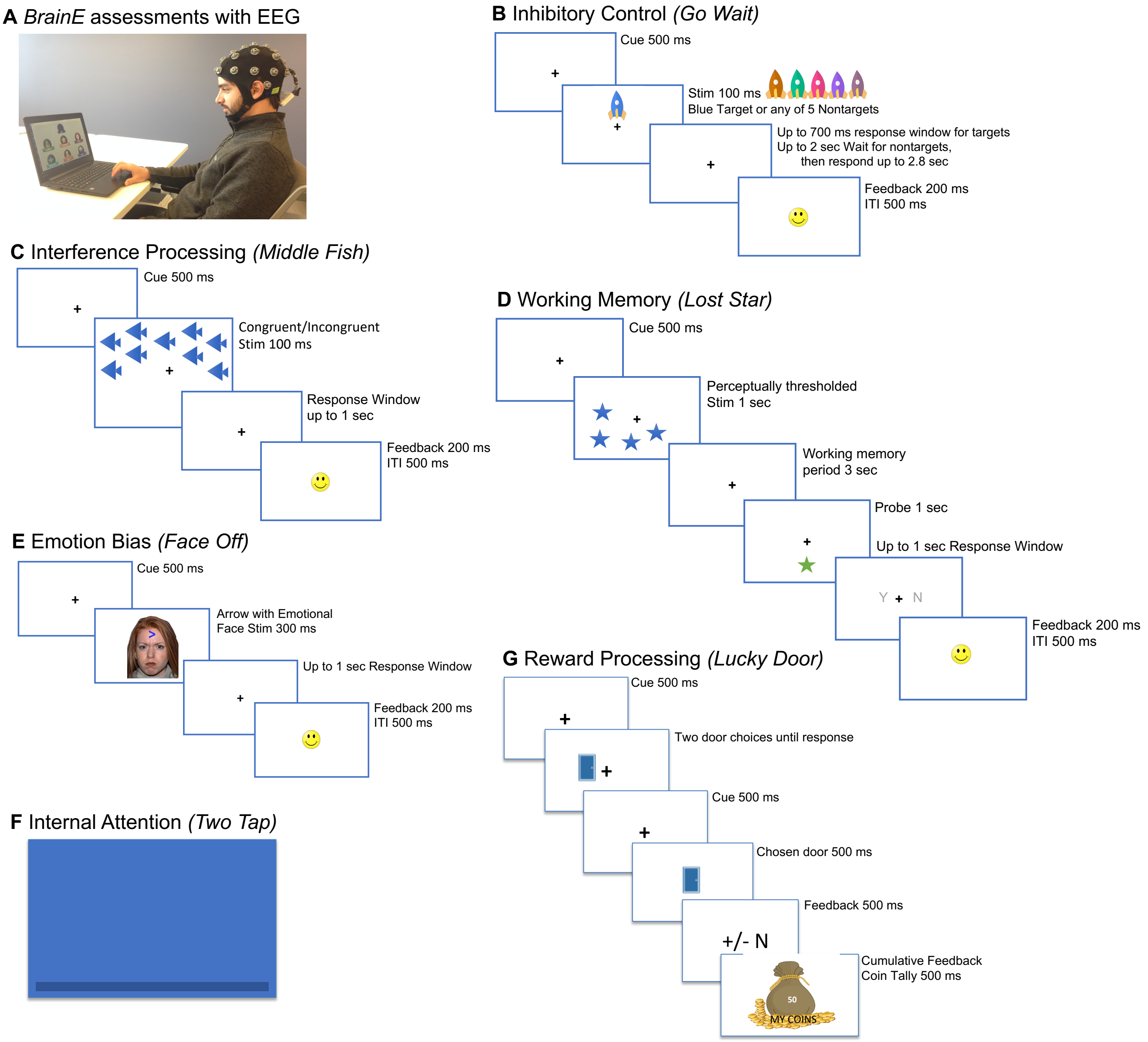

Supplementary Figure 1. Schematic layout of the six neuro-cognitive assessment tasks in which all participants engaged on day 1, day 15, and day 30. A) Snapshot of computerized assessment with EEG; (B) Inhibitory control task; (C) Interference processing task; (D) Working memory task; (E) Emotion bias task; (F) Internal attention task with no-onscreen stimuli; and (G) Reward processing task.

Supplementary Table 1: Summary of 43 features used in the study for personalized mood prediction.

#	Feature Name	Feature Description
1	*anxious*	EMA-based 1-7 ratings of "How relaxed versus anxious do you feel right now?" acquired 4x per day alongside the depressed mood ratings
2	*Mean Breathing Time*	Mean breathing time of the 30-sec active stress assessment acquired 4x per day alongside the depressed mood ratings
3	*Consistency*	Consistency of breathing (1 – coefficient of variation) of the 30-sec active stress assessment acquired 4x per day alongside the depressed mood ratings
4	*past day fats*	Total fatty items consumed in the 24 hours prior to each depressed mood rating
5	*past day sugars*	Total sugary items consumed in the 24 hours prior to each depressed mood rating
6	*past day caffeine*	Total cups of caffeine consumed in the 24 hours prior to each depressed mood rating
7	*heart rate*	Smartwatch-based heart rate as the mean heart rate in the ±30 minute window around each depressed mood EMA
8	*ppg_std*	Heart Rate Variability from the Tizen® Photoplethysmography data as the standard deviation within the ±15 minute window around each depressed mood EMA
9	*cumm_step_count*	Cumulative step count taken as the mean value from the past 12 hours of each depressed mood rating
10	*cumm_step_calories*	Cumulative step calories burnt taken as the mean value from the past 12 hours of each depressed mood rating
11	*cumm_step_distance*	Cumulative step distance taken as the mean value from the past 12 hours of each depressed mood rating
12	*cumm_exercise_calories*	Cumulative exercise calories burnt taken as the mean value from the past 24 hours of each depressed mood rating
13	*cumm_exercise_duration*	Cumulative exercise duration taken as the mean value from the past 24 hours of each depressed mood rating
14	*prev_night_sleep*	Number of hours of sleep the previous night of each depressed mood rating
15	*time_of_day*	Time of the day when a particular depressed mood rating was taken: (6:00, 10:00], (10:00, 14:00], (14:00, 18:00], (18:00, 23:59]
16	*GW Consistency*	Consistency of responses in the *Go Wait* inhibitory control task implemented at pre-, mid-, post- 30-day EMA monitoring
17	*GW Efficiency*	Performance Efficiency in the *Go Wait* inhibitory control task implemented at pre-, mid-, post- 30-day EMA monitoring
18	*MF Consistency*	Consistency of responses in the *Middle Fish* interference processing task implemented at pre-, mid-, post- 30-day EMA monitoring
19	*MF Efficiency*	Performance Efficiency in the *Middle Fish* interference processing task implemented at pre-, mid-, post- 30-day EMA monitoring
20	*LS Span*	Working memory span 1-8 in the *Lost Star* working memory task implemented at pre-, mid-, post- 30-day EMA monitoring
21	*LS Consistency*	Consistency of responses in the *Lost Star* working memory task implemented at pre-, mid-, post- 30-day EMA monitoring
22	*LS Efficiency*	Performance Efficiency in the *Lost Star* working memory task implemented at pre-, mid-, post- 30-day EMA monitoring
24	*FO Consistency*	Consistency of responses in the *Face Off* emotion bias task implemented at pre-, mid-, post- 30-day EMA monitoring
25	*FO Efficiency*	Performance Efficiency in the *Face Off* emotion bias task implemented at pre-, mid-, post- 30-day EMA monitoring

26	*Mean Breathing Time TT*	Mean breathing time on the *Two Tap* internal attention to breath task implemented at pre-, mid-, post- 30-day EMA monitoring
27	*Consistency TT*	Consistency of breathing on the *Two Tap* internal attention to breath task implemented at pre-, mid-, post- 30-day EMA monitoring
28	*LD_GL_bias*	Bias towards frequent gain vs. loss in the *Lucky Door* reward task implemented at pre-, mid-, post- 30-day EMA monitoring
29	*LD_RareG_diff*	Preference for rare gain choices when these choices have greater vs. equal expected value in the *Lucky Door* reward task implemented at pre-, mid-, post- 30-day EMA monitoring
30	*gw_leftDLPFC*	Neural activity in the left DLPFC brain region evoked to the *Go Wait* inhibitory control task implemented at pre-, mid-, post- 30-day EMA monitoring
31	*gw_dACC*	Neural activity in the dACC brain region evoked to the *Go Wait* inhibitory control task implemented at pre-, mid-, post- 30-day EMA monitoring
32	*mf_leftDLPFC*	Neural activity in the left DLPFC brain region evoked to the *Middle Fish* interference processing task implemented at pre-, mid-, post- 30-day EMA monitoring
33	*mf_dACC*	Neural activity in the dACC brain region evoked to the *Middle Fish* interference processing task implemented at pre-, mid-, post- 30-day EMA monitoring
34	*ls_leftDLPFC*	Neural activity in the left DLPFC brain region evoked to the *Lost Star* working memory task implemented at pre-, mid-, post- 30-day EMA monitoring
35	*ls_dACC*	Neural activity in the dACC brain region evoked to the *Lost Star* working memory task implemented at pre-, mid-, post- 30-day EMA monitoring
36	*fo_leftDLPFC*	Neural activity in the left DLPFC brain region evoked to the *Face Off* emotion bias task implemented at pre-, mid-, post- 30-day EMA monitoring
37	*fo_dACC*	Neural activity in the dACC brain region evoked to the *Face Off* emotion bias task implemented at pre-, mid-, post- 30-day EMA monitoring
38	*TT_left DLPFC*	Neural activity in the left DLPFC brain region during the *Two Tap* internal attention to breath task implemented at pre-, mid-, post- 30-day EMA monitoring
39	*TT_dACC*	Neural activity in dACC brain region during the *Two Tap* internal attention to breath task implemented at pre-, mid-, post- 30-day EMA monitoring
40	*GLbias_left DLPFC*	Neural activity in the left DLPFC brain region corresponding to bias for frequent gains vs. losses on the reward task implemented at pre-, mid-, post- 30-day EMA monitoring
41	*GLbias_dACC*	Neural activity in the dACC brain region corresponding to bias for frequent gains vs. losses on the reward task implemented at pre-, mid-, post- 30-day EMA monitoring
42	*diff_rareLG_left DLPFC*	Neural activity in the left DLPFC brain region corresponding to choices made on the reward task with a contrast of expected values, implemented at pre-, mid-, post- 30-day EMA monitoring
43	*diff_rareLG_dACC*	Neural activity in the dACC brain region corresponding to choices made on the reward task with a contrast of expected values, implemented at pre-, mid-, post- 30-day EMA monitoring

Supplementary Methods

ML Pipeline: Nested CV. This algorithm is used to perform hyperparameter tuning and model selection by attempting to overcome the problem of overfitting the training dataset[85,86]. It involves treating model hyperparameter tuning as part of the model itself and evaluating it within the broader k-fold CV procedure for evaluating models for comparison and selection. The k-fold CV procedure for model hyperparameter optimization is nested inside the k-fold CV procedure for model selection. Given that the procedure uses two CV loops it is also called double CV. Typically, the k-fold CV procedure involves fitting a model on all folds but one and evaluating the fit model on the holdout fold. Nested CV estimates the generalization error of the underlying model and its hyperparameter search. By choosing the parameters that maximize non-nested CV, biases the model to the dataset, yielding an overly-optimistic score. Model selection without nested CV uses the same data to tune model parameters and evaluate model performance. Information may thus "leak" into the model and overfit the data. The magnitude of this effect is primarily dependent on the size of the dataset and the model's stability. To avoid this problem, nested CV effectively uses a series of train/validation/test set splits. In the inner loop, the score is approximately maximized by fitting a model to each training set and then directly maximized in selecting hyperparameters over the validation set. In the outer loop, generalization error is estimated by averaging test set scores over several dataset splits. Under this procedure, the hyperparameter search does not have an opportunity to overfit the dataset as it is only exposed to a subset of the dataset provided by the outer CV procedure. This reduces, if not eliminates, the risk of the search procedure overfitting the original dataset and should provide a less biased estimate of a tuned model's performance on the dataset. In this way, the performance estimate includes a component properly accounting for the error introduced by overfitting the model selection criterion. As far as model selection is concerned, a nested CV scheme produces k-surrogate “best” models out of which one has to be chosen based on a stability measure, which will then be refitted onto the dataset for intervention purposes.

A repeated k-fold CV repeats k-fold n times with different randomization in each repetition. This ensures the robustness and stability of the model. We used a repeated 4-fold CV scheme with ten repeats as the inner CV strategy and a simple 4-fold CV scheme as the outer CV strategy for the overall nested CV scheme. The overall best model selection of the ten surrogate best models that the inner CV scheme produces is made and refitted over a repeated 4-fold CV. Now, every ML hyperparameter optimization and training method needs a criterion over which optimization takes place. It is imperative to choose a metric per the data and the target which the ML model is trying to predict. We have not used traditional regression optimization such as mean absolute error and mean squared error for prediction purposes due to the sole reason that these metrics are not adequately sensitive in predicting depression states between Likert scale categories of 1 to 7. We instead used the mean Tweedie deviation function of negative power[100–102]. The Tweedie distributions are defined as a subfamily of exponential dispersion models, with a special mean-variance relationship. While the exact details on finding mean Tweedie deviance are outside the scope of this paper, we care about how it penalizes ML models' output with respect to the target variable that is the depressed mood state. For instance, let us consider a fitted ML model is used to predict test data points. Please refer to the following table that compares the penalization of a mean Tweedie function of negative power and a mean absolute deviation function for a hypothetical set of predicted and actual target variables' values.

Sr No.	Actual Values	Predicted Values	Mean Absolute Error	Mean Tweedie Deviation
1	[1, 2, 3, 4, 5, 6, 7]	[1, 2, 3, 4, 5, 6, 7]	0.0	1.54e-15
2	[1, 2, 3, 4, 5, 6, 7]	[1, 2, 3, 4, 5, 6, 6]	0.14	0.36
3	[1, 2, 3, 4, 5, 6, 7]	[1, 2, 3, 4, 5, 6, 5]	0.29	1.36
4	[1, 2, 3, 4, 5, 6, 7]	[1, 2, 3, 4, 5, 5, 5]	0.43	1.69
5	[1, 2, 3, 4, 5, 6, 7]	[5, 5, 5, 5, 5, 5, 5]	1.86	10.08
6	[1, 2, 3, 4, 5, 6, 7]	[1, 2, 3, 4, 5, 6, 3]	0.57	4.73
7	[1, 2, 3, 4, 5, 6, 7]	[2, 3, 4, 5, 6, 7, 7]	0.85	1.70
8	[1, 2, 3, 4, 5, 6, 7]	[1, 1, 2, 3, 4, 5, 6]	0.85	1.63
9	[1, 2, 3, 4, 5, 6, 7]	[3, 4, 5, 6, 7, 7, 7]	1.57	6.22

Notice from the examples shown above that the mean Tweedie deviance function of negative power is more sensitive to small changes in mood states and is better at penalizing the model for incorrect outputs than the mean absolute function. Using a mean Tweedie function is better suited to work as an optimization metric during hyperparameter tuning and model training.

ML Pipeline: ML Strategies. We used seven ML strategies for each subject (Figure 1). The pipeline was implemented in Python and details for each strategy used including Elastic Net, Support Vector, Poisson Regressor and Ensemble methods including Random Forest, Gradient Boost, Adaptive (Ada) Boost, and finally, the Voting Regressor are provided below[103].

Elastic Net Regression is a linear regression with combined $l1$ and $l2$ priors as regularizers[104,105]. Elastic Net has two hyperparameters: α and $l1$-*ratio* (ρ). α is a constant that multiplies the penalty terms. α = 0 is equivalent to an ordinary least square. ρ is the mixing parameter, with 0 <= ρ <= 1. If ρ = 0, the penalty is an $l2$ penalty, and if ρ = 1, then it is an $l1$ penalty. For 0 < ρ < 1, the penalty is a combination of $l1$ and $l2$.

To summarize, it optimizes the following cost function:

$$\min_{w} \frac{1}{2n_{samples}} {||Xw - y||_2}^2 + \alpha\rho||w||_1 + \frac{\alpha(1-\rho)}{2} {||w||_2}^2$$

Support Vector Machines (SVMs) are a set of supervised learning methods for regression that are: a) effective in high dimensional spaces, b) effective in cases where the number of dimensions is comparable to the number of samples, c) only uses a subset of training points in the decision function (called support vectors), so it is also memory efficient and d) versatile as different kernel functions can be specified for the decision function[106,107]. It has three significant hyperparameters kernel, C, and γ. Kernel refers to the type of kernel, namely linear, polynomial, or rbf, that is to be used, C is inversely related to the strength of regularization, and γ is the kernel coefficient.

Poisson Regressor is a Generalized Linear Model with a Poisson distribution[108,109]. The only hyperparameter in the case of a Poisson Regressor is α which defines the amount of regularization.

Ensemble Learning methods are a set of non-linear ML techniques that combine the predictions of several base estimators built with a given learning algorithm to improve generalizability or robustness over a mono-estimator. Due to this enhanced generalizability, ensemble learning methods are usually more immune to overfitting than other methods. More importantly, ensemble learning methods are more transparent than most ML methods as one can establish a relationship between the input and output almost as easily as in the case of a linear model.

There are two families of ensemble methods:
- Averaging Methods: the driving principle is to build several estimators independently and then to average their predictions. On average, the combined estimator is usually better than any of the single base estimator because its variance is reduced.
- Boosting Methods: the base estimators are built sequentially, and one tries to reduce the bias of the combined estimator. The motivation is to combine several weak models to produce a powerful ensemble.

In **Random Forests**, each tree in the ensemble is built from a sample drawn with replacement (i.e., a bootstrap sample) from the training set[110,111]. Furthermore, when splitting each node during a tree's construction, the best split is found from all input features. The trainable hyperparameters include the number of trees/estimators, maximum depth of decision trees, maximum no. of features used for predicting dependent variable, the minimum number of samples required to split an internal node, and the minimum number of samples required to be at a leaf node. The purpose of these two sources of randomness is to decrease the variance of the forest estimator. Indeed, individual decision trees typically exhibit high variance and tend to overfit. The injected randomness in forests yields decision trees with somewhat decoupled prediction errors. By taking an average of those predictions, some errors can cancel out. Random forests achieve a reduced variance by combining diverse trees, sometimes at the cost of a slight increase in bias. In practice, the variance reduction is often significant hence yielding an overall better model.

The core principle of **Ada Boost Trees** is to fit a sequence of weak learners (i.e., models that are only slightly better than random guessings, such as small decision trees) on repeatedly modified versions of the data[108-109]. The predictions from all of them are then combined through a weighted majority vote (or sum) to produce the final prediction. The data modifications at each so-called boosting iteration consist of applying weights to each of the training samples. Initially, those weights are all set to 1/N, so that the first step trains a weak learner on the original data. For each successive iteration, the sample weights are individually modified, and the learning algorithm is reapplied to the reweighted data. At a given step, those training examples that were incorrectly predicted by the boosted model induced at the previous step have their weights increased, whereas the weights are decreased for those that were predicted correctly. As iterations proceed, examples

that are difficult to predict receive ever-increasing influence. Each subsequent weak learner is thereby forced to concentrate on the examples missed by the previous ones in the sequence.

Gradient Boosted Decision Trees (GBDT) is a generalization of boosting to arbitrary differentiable loss functions[109–111]. GBDT is an accurate and effective off-the-shelf procedure used for regression and classification problems in diverse areas, including Web search ranking and ecology. Each tree's size can be controlled either by setting the tree depth or setting the number of leaf nodes. The learning rate is a hyperparameter in the range (0.0, 1.0] that controls overfitting via shrinkage.

Voting Regressors can be considered a composite ensemble learning method[112]. The idea behind the Voting Regressor is to combine conceptually different ML regressors and return the average predicted values. Such a regressor can help a set of different ML models to balance out their individual weaknesses. The only hyperparameter in case of a voting regressor is weights. These weights are used to take a weighted average of the different ML models fed into a voting regressor. There are different strategies for assigning weights, such as giving more weight to one model than the other, giving different weights to the ensemble and non-ensemble learning models, and giving equal weights to all the models.

Supplementary Table 2

Correlations between Actual and Predicted Depression States in Each Subject				
**Subject ID**	**coefficient**	**p-value**	**low**	**high**
**P-1**	0.4512	7.518E-07	0.2884	0.5887
**P-10**	0.3848	1.110E-05	0.2230	0.5260
**P-12**	0.3559	2.786E-04	0.1715	0.5163
**P-14**	0.4470	8.041E-03	0.1282	0.6821
**P-15**	0.5601	9.496E-09	0.3993	0.6874
**P-18**	-0.3253	6.048E-02	-0.5977	0.0145
**P-19**	0.7504	2.061E-22	0.6586	0.8202
**P-20**	0.3395	5.292E-03	0.1062	0.5374
**P-21**	0.6159	8.956E-14	0.4903	0.7165
**P-23**	0.4439	2.974E-06	0.2729	0.5876
**P-24**	0.0987	2.836E-01	-0.0820	0.2731
**P-26**	0.1922	4.233E-02	0.0069	0.3648
**P-28**	0.4642	6.091E-07	0.2992	0.6023
**P-29**	0.2878	1.914E-02	0.0491	0.4953
**OVERALL**	0.6661	2.895E-167	0.6347	0.6953

Spearman rank correlation coefficients with p-values and 95% confidence interval bounds are reported for correlations across time between actual depressed states and those predicted from the personalized ML pipeline. Correlations were performed in each subject separately as well as overall by concatenating the data across all subjects. All correlations were significant except in P-18 and P-24.
